# Computer design of microfluidic mixers for protein/RNA folding studies

**DOI:** 10.1371/journal.pone.0198534

**Published:** 2018-06-20

**Authors:** Venkatesh Inguva, Sagar V. Kathuria, Osman Bilsel, Blair James Perot

**Affiliations:** 1 Department of Mechanical and Industrial Engineering, University of Massachusetts Amherst, Amherst, Massachusetts, United States of America; 2 Department of Biochemistry and Molecular Pharmacology, University of Massachusetts Medical School, Worcester, Massachusetts, United States of America; Massachusetts Institute of Technology, UNITED STATES

## Abstract

Kinetic studies of biological macromolecules increasingly use microfluidic mixers to initiate and monitor reaction progress. A motivation for using microfluidic mixers is to reduce sample consumption and decrease mixing time to microseconds. Some applications, such as small-angle x-ray scattering, also require large (>10 micron) sampling areas to ensure high signal-to-noise ratios and to minimize parasitic scattering. Chaotic to marginally turbulent mixers are well suited for these applications because this class of mixers provides a good middle ground between existing laminar and turbulent mixers. In this study, we model various chaotic to marginally turbulent mixing concepts such as flow turning, flow splitting, and vortex generation using computational fluid dynamics for optimization of mixing efficiency and observation volume. Design iterations show flow turning to be the best candidate for chaotic/marginally turbulent mixing. A qualitative experimental test is performed on the finalized design with mixing of 10 M urea and water to validate the flow turning unsteady mixing concept as a viable option for RNA and protein folding studies. A comparison of direct numerical simulations (DNS) and turbulence models suggests that the applicability of turbulence models to these flow regimes may be limited.

## Introduction

The advent of microfluidics has popularized hydrodynamic focusing [1] to initiate the folding or unfolding process of biological macromolecules such as proteins and RNA *via* laminar diffusion into a dilution buffer solution. The approach achieves microsecond mixing times with sample consumption of as little as femtomoles [2]. Chaotic mixers improve the performance of laminar mixers by using geometry [3] to add stretching and folding [4,5] of the solutions being mixed (e.g., an unfolded protein solution diluted with refolding buffer), resulting in an increase of the surface area and reduction of the diffusion length to enable faster mixing under certain conditions [3]. Chaotic mixers have larger channels because mixing occurs all throughout the channel instead of just in the focused region as in laminar mixers. Turbulent mixers on the other hand use the significant disorder in the fluid to initiate kinetics by changing the solution conditions of biological macromolecules. Examples of turbulent mixers are capillary mixers [6] and T-mixers [7].

These mixing approaches use different physics to achieve mixing. Laminar mixers rely on molecular diffusion to perform mixing. Chaotic mixers add flow velocity transverse to the flow direction to increase fluid parcel movement thereby increasing mixing. Turbulent mixers use significant disorder in the fluid itself to rapidly transport fluid parcels within the fluid to do mixing.

The choice of microfluidic mixer to use for initiating kinetics is influenced by the spectroscopic technique used for probing the reaction of interest. The observation volume is significantly different for chaotic/turbulent mixers compared to laminar mixers and this in turn also affects signal-to-noise and sample concentrations. The hydrodynamic focusing aspect (0.1 to 1 μm in width) of laminar mixers requires the use of highly focused beams and high concentrations [8] (100 to 500 μM) to obtain good signal-to-noise ratios for intrinsic fluorescence and transmission based spectroscopy. Turbulent mixers require lower concentrations [9–11] (3 to 10 μM) and provide larger observation channels [12] (50 to 100 μm in width). Laminar mixers achieve 90% mixing efficiency in less than 10 μs [8,13] while turbulent mixing achieves 99% mixing efficiency in as little as 30 μs [12]. Laminar mixers use significantly less biomolecule sample than turbulent mixers (femtomoles [2] to micromoles [14]).

This paper presents a computer-aided approach to designing mixers which merges the diffusion initiated kinetics of hydrodynamic focusing with the significant disorder of bulk fluid motion associated with turbulently initiated kinetics, capturing the advantages of both classes of mixers while attempting to mitigate the disadvantages (i.e., reducing sample consumption while maintaining large observation channels with reasonable mixing times). Specifically, we model various chaotic/marginally turbulent mixing concepts such as flow turning, flow splitting, and vortex generation using urea solution mixed with buffer, which is modelled as water mixing with water. Additionally, one of the limitations in computer-aided design of mixers operating at the onset of turbulence or chaotic regimes is that the applicability of turbulence models, which are intended for fully turbulent flows at high Reynolds numbers, is not clear. We address this outstanding question by performing simulations of mixing using various turbulence models and comparing the results with direct numerical simulations, which is model independent. The comparison allows us to critically evaluate the applicability of various turbulence models for use in mixer design in these flow regimes.

## Methods

This work uses an open source computational fluid dynamics (CFD) tool so other research groups can emulate the methodology. The parallel CFD solver twoLiquidMixingFoam from the OpenFoam [15] software suite was used. CAD design of the mixers was done in FreeCAD [16] and mesh generation of the fluid volume was done using the commercial software ICEM CFD [17]. Hexahedral elements were chosen over tetrahedral elements to minimize cell count and improve solver stability. Free meshing software such as Salome Platform [18] and the OpenFoam BlockMesh [15] utility could also be used for mesh generation. To visualize and post-process the results from the solver, the open source program, Paraview [19] 4.3.1 was used. No modification of any software tools was necessary to perform the design process.

The mixing process was modelled as one stream of water (containing unfolded protein and urea) mixing with two other streams of water (buffer). Experimentally, the concentration of urea solution[20] is typically 6 to 8 M and the viscosity[21] is ~30% higher than that of water. This viscosity difference is not large enough to significantly affect macroscopic mixing. As discussed in the turbulence models in design section, the turbulence models which work for this problem predict turbulent viscosities that are ~300% higher than the fluid viscosity. In the bounded scalar transport section, turbulent viscosity predicts the macroscopic mixing from a modelling perspective. As such, relatively small fluid viscosity differences would not impact macroscopic mixing significantly. The solver has the capability to model two fluids with different viscosities and densities. However, variable density and relatively low difference in viscosity between urea solution and water is not critical to the mixing physics that occurs in this class of mixers and is therefore not used.

### Consideration of solution additives on mixing

An assumption in our simulations is that the protein, buffer reagents and other small molecules added to the solutions do not perturb the mixing process in a measurable way. We present several lines of reasoning to justify this assumption. Experimentally [22], protein is added to the urea solution to create the unfolded protein solution. For both the unfolded protein solution and the dilution buffer a standard buffer is used, such as 10 mM potassium phosphate in water. The concentration of protein in the urea solution is typically in the micromolar range and not significant enough to change the viscosity of the protein solution, which will exhibit the viscosity of the urea solution to which the protein was added. Similarly, the buffer solution will exhibit the viscosity of water because the concentration of potassium phosphate is not significant enough to change the viscosity of water. Mixing in this class of mixers is not a molecular process but a macroscopic process performed by large fluid parcels mixing with each other. Different biological macromolecules may exhibit different kinetics during mixing, but they themselves do not affect the macroscopic mixing. Although the biomolecule has no effect on the macroscopic mixing in this class of mixers it is possible that the flow might perturb the conformation or oligomeric state of very large macromolecules (e.g., long fibrils, large megadalton complexes). Experiments on relatively small complexes (molecular weight <150 kDa) suggest this is not a significant issue, but the behavior of larger complexes or highly disordered complexes is an open question.

As such, it is necessary to model the fluids being mixed (i.e., the urea solution and water) rather than the relatively low concentrations of biological macromolecules and potassium phosphate (or other buffers) in the buffer that do not affect the fluid properties significantly. As discussed further below in our treatment of bounded scalar transport, the Schmidt number provides the means to model different solutions for the protein (i.e., other than urea). This study only considers urea solution as the protein inlet solution. Furthermore, this study does not consider nanoscale particles and entities changing the fluid properties described in studies related to nanofiber fabrication by bubble electrospinning [23,24].

### Mixer concepts

**Fig 1:**
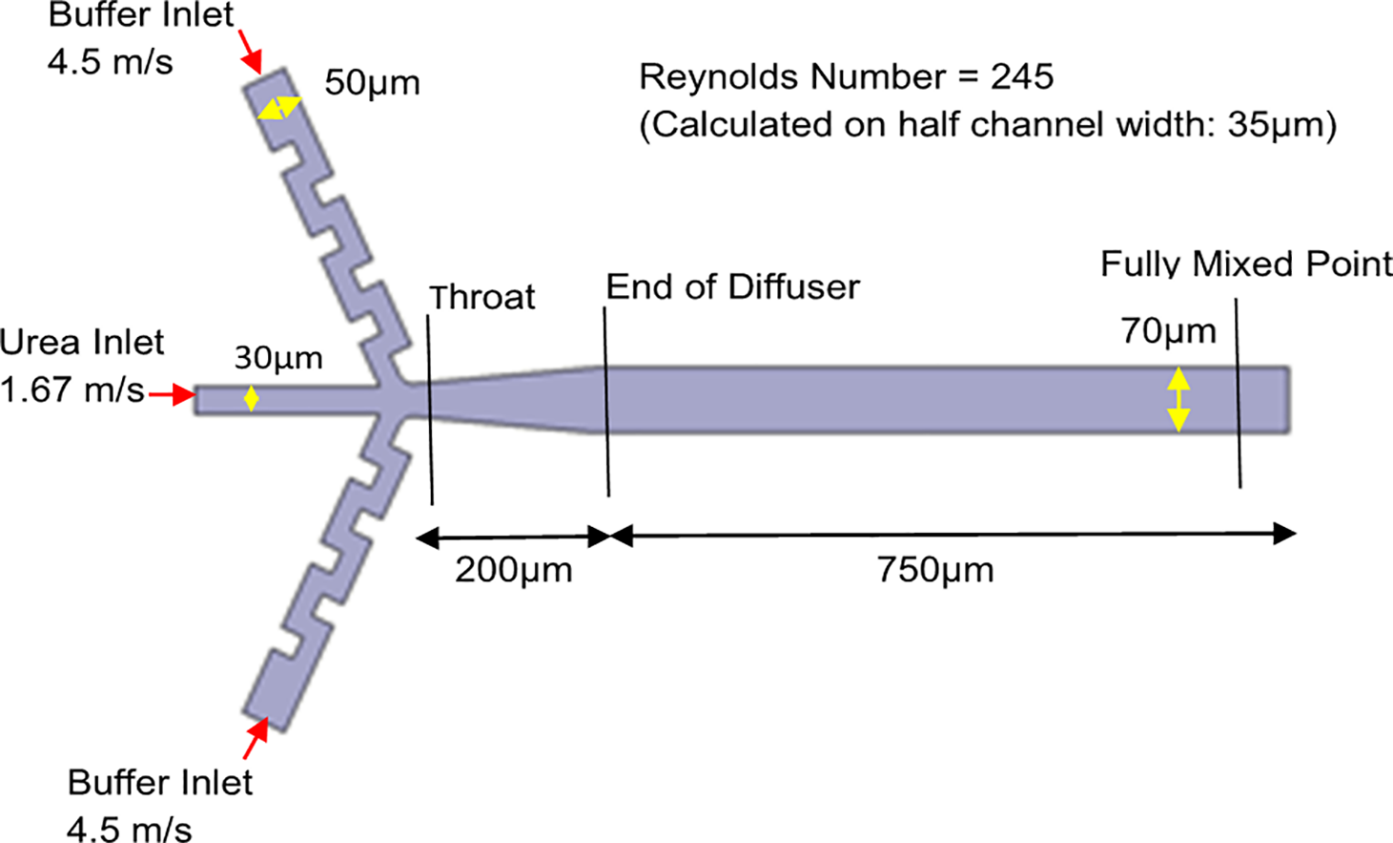
Operating conditions shown on first design concept in Fig 2A, but applicable to all design concepts except that in Fig 2B.

**Fig 2:**
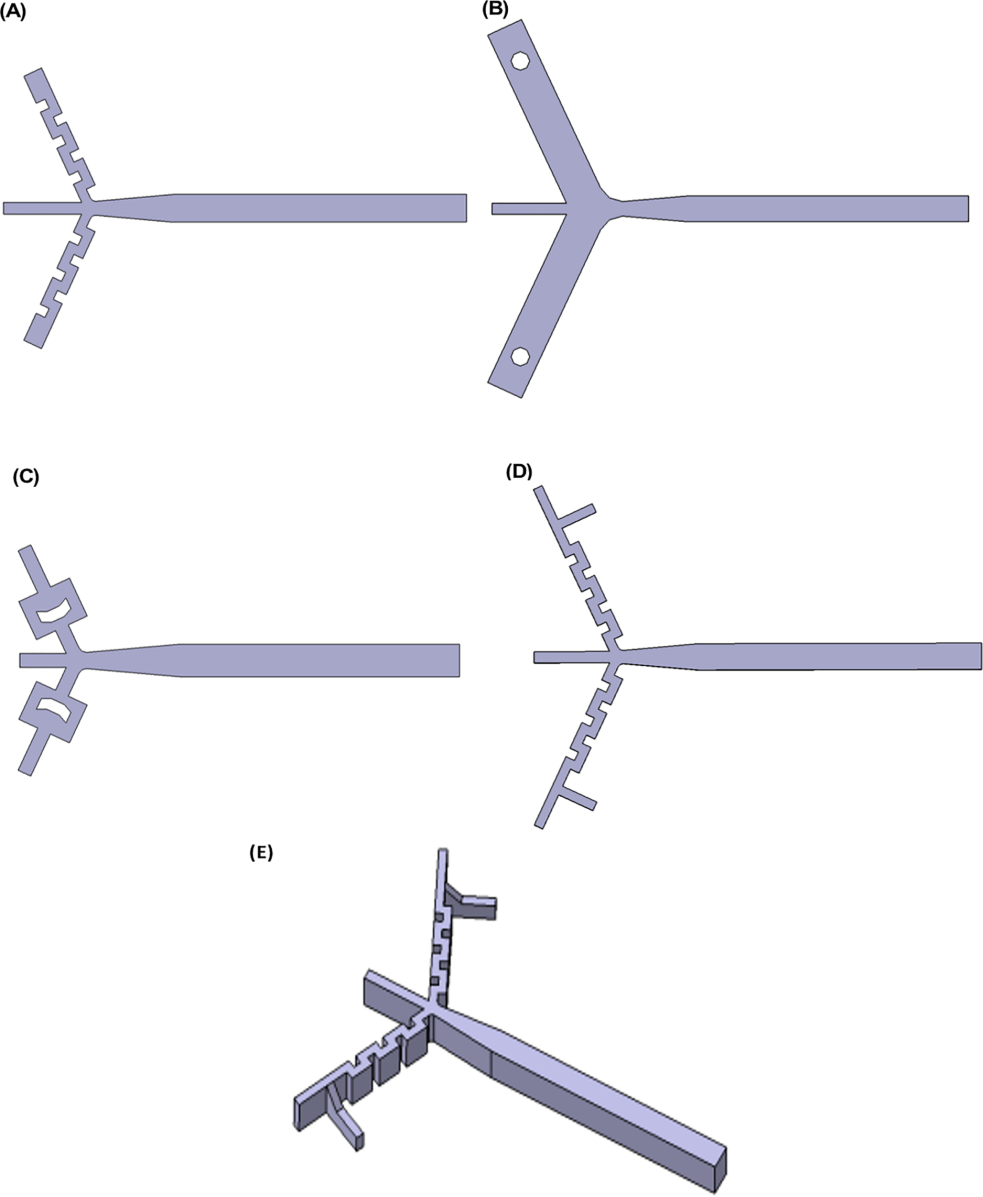
Five unsteady/turbulent mixer designs tested in this work. (A) flow-turning inlets, (B) vortex shedding inlets (side channels are twice the width of other concepts, centre channel is same width as other channels, buffer solution flow rate in side channels of 9 m/s), (C) flow-splitting inlets, (D) T-junction flow-turning inlets, (E) T-junction swirled flow-turning inlets.

This class of mixers operate with a 1:10 protein (or RNA) solution to buffer solution dilution ratio and at a total flow rate of 3 mL/min at the exit. A diffuser is used to expand the mixied urea solution flow from a channel width of 30 microns to 70 microns so as to provide a larger observation area (especially for small-angle x-ray scattering) and in order to slow the fluid down in the measurement section to increase the longest observable time. The operating conditions of the first concept are shown in Fig 1. The Reynolds number(Re), is 245, based on a half-channel width of 35 μm and the average velocity of 7 m/s in the channel calculated from conservation of mass based on inlet speeds. All design concepts have the same inlet flow rates and dimensions except the concept in Fig 2B, which has four times the flow rate of buffer solution and twice the buffer inlet width to encourage vortex shedding to occur. The end of the diffuser, where the expansion becomes a straight channel, is the beginning of the first measurement point for the spectroscopic probe and is therefore chosen as the point of reference to study the urea concentration levels. It should be noted that a Reynolds number of 245 is laminar only in simple channel flow. This is not the case for the mixing concepts presented and chaos/turbulence can be achieved with the flow configurations presented. This study also did not consider non-rectangular channels because spectroscopic techniques such as SAXS require flat observation channels to prevent refraction from occurring. It is also relatively easier to manufacture high aspect ratio rectangular channels using laser etching [25].

Fig 2 shows the various design concepts tested by the simulation approach. A common feature of the designs is that turbulence is generated in the dilution buffer prior to the flow merging with the central urea containing solution that is being diluted. This design feature is expected to minimize potential shearing of large biomolecular complexes. It was also desired that the flow in the mixing region be symmetric to ensure even distribution of urea transverse to the direction of the flow. Placing turbulence generators after the urea solution and buffer solution meet will induce asymmetry in flow profile as a result of the geometry causing urea to pool to a particular region transverse to the flow direction. One design constraint is for the mixing to be complete within 50 μs of the urea meeting the buffer. The quicker the mixing the more precise the timing of the kinetic reaction (e.g., folding of the protein) can be determined. Another design constraint is to minimize sample consumption, as protein and RNA samples can be expensive to obtain. An additional design constraint is for the observation channel to be a minimum of 50 microns wide for interfacing with optical and x-ray spectroscopy techniques (e.g., fluorescence and small-angle x-ray scattering). A final design consideration was to ensure symmetry in geometry so that quasi-symmetric fluid flow develops, providing a consistent measurement stream for subsequent detection of the kinetics.

Fig 2A to 2C show the various mixing concepts studied. These concepts include flow turning (Fig 2A), vortex shedding (Fig 2B) and flow splitting (Fig 2C). Simulation results presented below show flow turning to be the best candidate for mixing. To improve on the flow turning concept, two additional flow turning concepts were explored in Fig 2D and 2E. In Fig 2D, the inlets were split into two, 90° apart, to induce further instabilities. In Fig 2E this splitting was only at half height to induce flow swirl. All the simulated geometries are 100 microns in depth. Fig 2A to 2D are two-dimensional designs extruded in depth while Fig 2E is a three-dimensional design.

### Incompressible Reynolds Averaged Navier-Stokes (RANS)

TwoLiquidMixingFoam allows different levels of turbulence modelling to be performed to account for the unsteadiness and small scale unsteady chaotic 3D fluid motion. It can solve for the mean flow using Reynolds Averaged Navier-Stokes (RANS) equations, or use a large eddy simulation (LES) subgrid scale model, or use no model at all and perform direct numerical simulation (DNS). This work evaluates the RANS and DNS options. LES will perform between these two options. A RANS model requires the least computational resources [26] but incurs significant model uncertainty for this type of problem. DNS is much more expensive to compute but is free from any modelling errors in the fluid flow, but not the mixing model.

Conservation of mass for incompressible flow is given by the following equation, where *U* is the velocity.

$$\frac{\partial \langle U_{i}\rangle }{\partial x_{i}}=0$$ (1)

The mean-momentum equation is given by the following equation, where *p* is the pressure and *ν* is the visocity.

$$\frac{\partial \langle U_{i}\rangle }{\partial x_{i}}+\langle U_{i}\rangle \frac{\partial \langle U_{i}\rangle }{\partial x_{i}}=\frac{\partial }{\partial x_{j}}\left[\nu (\frac{\partial \langle U_{i}\rangle }{\partial x_{j}}+\frac{\partial \langle U_{j}\rangle }{\partial x_{i}})-\frac{\langle p\rangle \delta_{ij}}{\rho }-\langle u_{i}u_{j}\rangle \right]$$ (2)

The above equation describes the momentum equation for Navier-Stokes with the addition of the Reynolds Stress Tensor 〈*u*_*i*_*u*_*j*_〉 yielding the RANS equation. Using the turbulent viscosity hypothesis [26], the Reynolds Stress Tensor can be modelled as follows, where *ν*_*T*_ is the turbulent viscosity and *k* is the turbulent kinetic energy.

$$\langle u_{i}u_{j}\rangle =\frac{2}{3}k\delta_{ij}-\nu_{T}\left(\frac{\partial \langle U_{i}\rangle }{\partial x_{j}}+\frac{\partial \langle U_{j}\rangle }{\partial x_{i}}\right)$$ (3)

For RANS computations, the values of turbulent kinetic energy *k* and turbulent viscosity *ν*_*T*_ are modelled using two additional partial differential equations (the *k* − *ω* SST model). For incompressible flow the turbulent kinetic energy term in Eq 3 is sometimes absorbed into the pressure term when Eq 3 is combined with Eq 2.

For the DNS simulations the Reynolds Stress Tensor becomes zero because all the flow time scales and spatial scales are accurately computed (so there are no fluctuations, and their average value is zero).

### *k* – *ω* Shear Stress Transport turbulence model

For this work the primary model used is the *k* − *ω* Shear Stress Transport [27] (SST) turbulence model. But three other two-equation models applicable to this flow situation are also evaluated. The SST model uses a blending function [27] to behave like *k* − *ε* in the free steam and *k* − *ω* in the boundary layer capturing some of the positives of both models while mitigating some of the negatives of each model. The detailed reasoning behind selecting this particular model from a design perspective is discussed in the Discussion section. The SST model equations to solve for *k* and *ν*_*T*_ in Eq 3 are.

$$\nu_{T}=\frac{\alpha_{1}k}{max(\alpha_{1}\omega ,\;SF_{2})}$$ (4)

$$\frac{\partial k}{\partial t}+\langle U_{j}\rangle \frac{\partial k}{\partial x_{j}}=P_{k}-\beta^{*}k\omega +\frac{\partial }{\partial x_{j}}\left[\left(\nu +\sigma_{k}\nu_{T}\right)\frac{\partial k}{\partial x_{j}}\right]$$ (5)

$$\frac{\partial \omega }{\partial t}+\langle U_{j}\rangle \frac{\partial \omega }{\partial x_{j}}=\alpha S^{2}-\beta \omega^{2}+\frac{\partial }{\partial x_{j}}\left[\left(\nu +\sigma_{\omega }\nu_{T}\right)\frac{\partial \omega }{\partial x_{j}}\right]+2(1-F_{1})\sigma_{\omega 2}\frac{1}{\omega }\frac{\partial \omega }{\partial x_{i}}\frac{\partial k}{\partial x_{i}}$$ (6)

In summary, Eq 5 defines the turbulent kinetic energy while Eq 6 determines the specific rate of dissipation. Eq 4 determines the turbulent viscosity determined from Eqs 5 and 6 and is used to close the Reynolds Stress Tensor in Eq 3. *F*_1_ and *F*_2_ are blending functions to blend the wall solution and free stream solution for *ν*_*T*_. *P*_*k*_ is turbulence production term. *S* is the strain-rate tensor. *α*_1_, β*, *β*, *σ*_*k*_, *σ*_*ω*_, *σ*_*ω*2_ are tuning constants for the turbulence model. The blending functions and tuning constants for this model are defined in reference 27. The other two-equation models have a very similar form that also requires solving one or two advection-diffusion-reaction partial differential equations like Eqs 5 and 6.

### Bounded scalar transport for mixing of two miscible liquids

TwoLiquidMixingFoam simulates the mixing between two miscible incompressible liquids by modelling the transport of one liquid phase into the other liquid phase. The fluids are assumed to be isothermal [28] and modelled in this work as water (containing urea) in the central inlet and water (buffer) from the two side inlets (See Fig 1). The transport and mixing are captured by tracing one stream of water with urea (marked with *α* = 1) entering from the central inlet as it mixes into the other two streams of water which are buffer (and marked *α* = 0) coming from the two side inlets. Mixing is computed by calculating the transport of the normalized urea concentration variable, *α*. The transport equation for *α* is given by the following equation [29].

$$\frac{\partial \alpha }{\partial t}+\langle U_{i}\rangle \frac{\partial \alpha }{\partial x_{i}}=\frac{\partial }{\partial x_{i}}\left[\left(\frac{\nu }{Sc}+\nu_{T}\right)\frac{\partial \alpha }{\partial x_{i}}\right]$$ (7)

The Schmidt number *Sc* of urea in water is 950 [30]. The diffusion constant *ν*/*Sc* quantifies the diffusion of urea into the buffer solution (large Sc means the regular molecular diffusion is very slow). The turbulent viscosity *ν*_*T*_ in Eq 7 models the much faster mixing due to fluid turbulence. Eq 7 represents the mixing/diffusion of urea into the buffer. The intrinsic assumption in this paper is that the urea solution, modelled with the properties of water, is diffusing and macroscopically mixing into water. The diffusion of urea into water is captured by the Schmidt number while the macroscopic mixing is captured by the turbulent viscosity *ν*_*T*_ term. *ν* is the viscosity of water. Hence, it is assumed that urea solution has the viscosity of water and the buffer has the viscosity of water.

Turbulence in these small sized mixers is fairly weak and only marginally turbulent. The nature of the turbulence under these conditions is not well understood and so the turbulent mixing may not be well captured by the existing turbulence models.

The *α* variable is bounded between 0 and 1 where 1 is the maximum level of urea at the inlet and 0 indicates only the presence of buffer solution and no urea. Any value between 0 and 1 indicates how much of the urea containing fluid has mixed with buffer solution. For the inflow conditions in this mixer (corresponding to a 10-fold dilution of the fluid in the central inlet), a perfectly mixed flow will have *α* = 0.1.

### Bernoulli’s equation and cavitation

Bernoulli’s Equation is used in the design process before any computations to determine the dimension of the throat of the mixer after the three inlets meet as shown in Fig 1. This region is where the velocity is the highest and is most prone to cavitation. A pressure of -1 atmosphere (gauge) is assigned to the throat and its area is determined based on the bulk flow velocity through the throat, the bulk flow velocity at the exit, and the pressure at the exit (0 gauge pressure). The equation to determine the minimum throat area, derived from Bernoulli’s Equation [31], is given by

$$A_{throat}=\sqrt{\frac{{A_{exit}}^{2}{Q_{exit}}^{2}\rho }{{Q_{exit}}^{2}\rho +(2\;atm){A_{exit}}^{2}}}$$ (8)

We recommend setting *A*_*throat*_ to be about 10% larger than predicted by Eq 8 and checking that solutions are free from cavitation (pressures lower than -100 kPa). *A*_*exit*_ is the area at the exit of the mixer. *Q*_*exit*_ is the total flow rate through the mixer which is also the flow rate at the exit of the mixer. *ρ* is the density of the fluid. At the throat, the conditions for Bernoulli’s Equation to hold are not perfectly satisfied, but the 10% margin added to *A*_*throat*_ is expected to compensate for this.

### Supercomputer, computation time and numerical schemes

The NSF supercomputer Stampede at the Texas Advanced Computing Center was used for all simulations. For RANS simulations, all concepts shown in Fig 2 were discretized with 2 million cells. The simulations required 64 cores provided by 4 nodes with each node consisting of 2 Xeon Intel 8-Core 64-bit E5-processors. The simulations ran for about 24 hours yielding 10 flows through time. DNS simulations required discretization of 50 million cells. The DNS simulations required 512 cores provided by 32 nodes consisting of the same processor. The DNS simulations ran for about 96 hours yielding 6 flows through time. An additional large memory node consisting of 1TB of random access memory was used to decompose and reconstruct the DNS cases for pre-processing and post-processing. RANS does not require as fine a mesh as DNS because scales of turbulence are modelled instead of resolved by the mesh like in DNS. Therefore, RANS meshes can be much coarser than DNS meshes. The numerical schemes in TwoLiquidMixingFoam are described as follows. Time stepping is done by an implicit Euler scheme. Divergences are done by a Gauss limitedLinear scheme. Gradients are done by a Gauss Linear scheme. Interpolation schemes are linear. Surface normal gradient schemes are corrected. All residuals for matrix inversion have their tolerances set to 1e-6 before iterations are complete.

## Results and discussion

All of the design concepts were tested using the k-ω SST turbulent model and one of the designs (Fig 2A) was tested using four turbulence models, described in the Turbulence Models in Design section, and compared with DNS. The concept in Fig 2A is designated as the reference design to which all designs will be compared. It is important to note that using a RANS turbulence model to resolve velocity and pressure fields only produces information about the average flow behavior. As such, the reader should be careful in drawing conclusions about how exactly the urea containing fluid is transported into the buffer solution from looking at RANS results. These simulations model and do not calculate the 3D unsteadiness that actually produces mixing.

**Fig 3:**
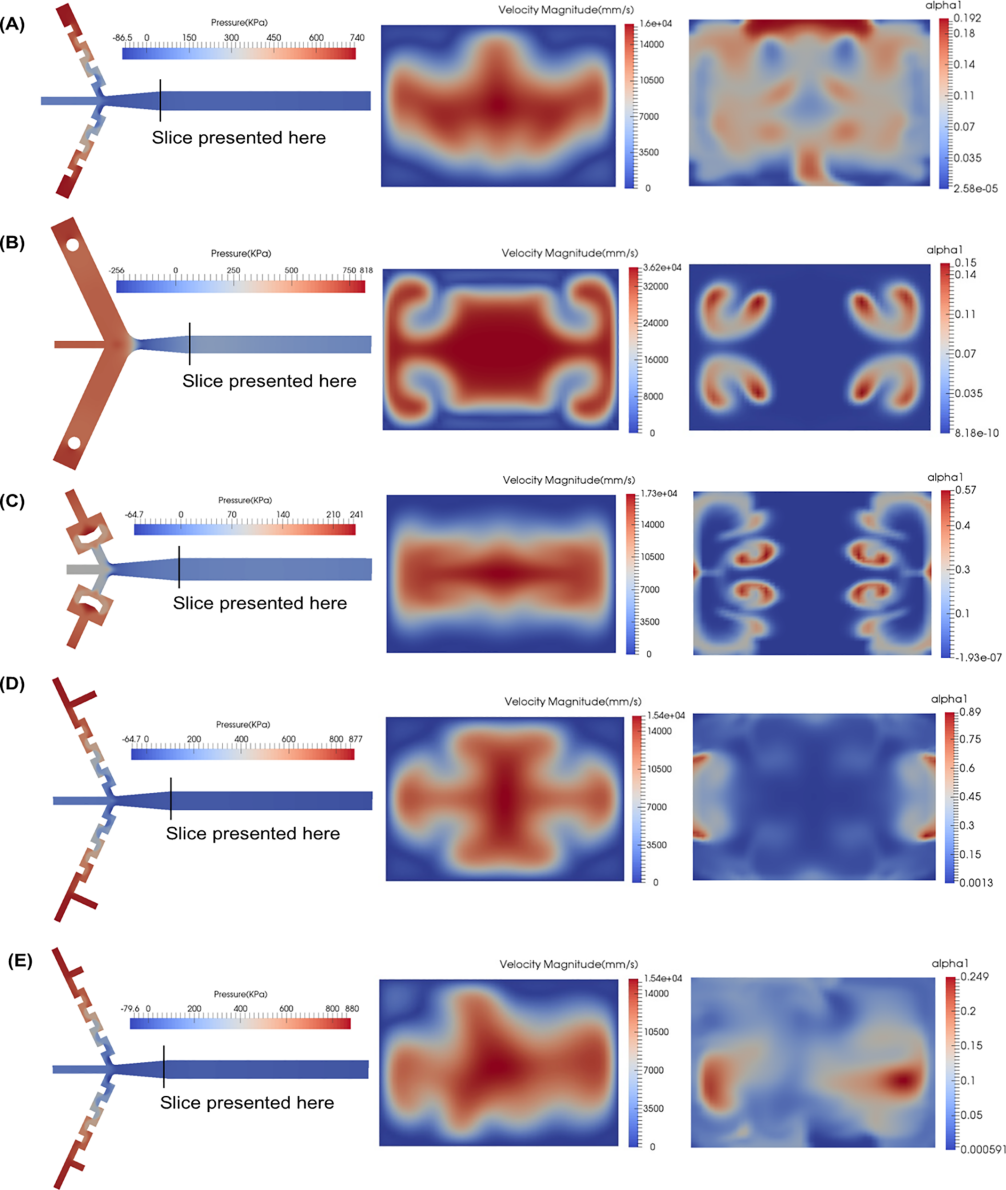
Pressure, velocity magnitude and *α* simulation results using the *k* − *ω* SST turbulence model. (A) Flow-turning inlets, (B) Vortex shedding inlets, (C) Flow-splitting inlets, (D) T-junction flow-turning Inlets, (E) T-junction swirled flow-turning Inlets.

Horizontal cross-sections of the velocity magnitude (speed) and *α* (urea concentration levels) are presented that are traverse to the direction of flow (and at the diffuser exit location) on the right of Fig 3. For pressure, it is more useful to study its development along the entire mixer. Cross sections of the pressure parallel to the flow for each design concept are presented on the left of Fig 3.

To analyze the mixing, *α* (or alpha1 as shown in the figures) is used to determine to what extent the urea containing fluid is transported into the buffer solution. What is sought is the most evenly spread profile. Since this is a 1:10 ratio dilution mixer, a fully mixed situation will have a uniform value of 0.1 throughout the entire mixer. From Fig 3A to 3C, it can be seen that the design concept in Fig 2A has the most uniform mixing of all of the initial concepts in Fig 2A to 2C. Interestingly, trying to improve on the flow turning concept of Fig 2A, by adding instabilities to the flow before flow turning as seen in Fig 3D and 3E, does not improve the mixing efficiency.

The velocity magnitude information shows a similar trend for all the mixers: fast fluid in the core of the mixer and slower flow near the mixer walls. Friction causes the fluid to be stationary on the walls. However, each mixer is showing differences in the specific patterns of the velocity magnitude. This is caused by differences in the large scale secondary motions within each mixer. Certain motions are better at fully mixing the fluids but it is impossible to guess which ones are most effective without simulating the mixing. Asymmetry in the velocity profiles in Fig 3A and 3E imply secondary motion is also occurring in the direction traverse to the bulk flow (out of the page).

The pressure plots shown in Fig 3 serve as the check for cavitation. The pressure plots of Fig 3A, 3C, 3D and 3E show that no cavitation is occurring (pressure never drops below -100 kPa). The throat area equation (Eq 8) is a useful design tool in this respect. The minimum pressure in Fig 3B drops to below -100 KPa because the flow rate is twice as high and the throat area was not increased in the design concept in Fig 2B. This design would cavitate.

The concepts in Fig 2A and 2E are good candidates to manufacture and implement on an experimental setup as seen from results in Fig 3A and 3E. The concept in Fig 2A shows some collection of urea containing fluid at the top wall, which the concept in Fig 2E mitigates by centering the collection of urea containing fluid to the middle of the channel at the cost of some mixing efficiency. The design in Fig 2A is much easier to manufacture.

Simulations using several different RANS models and comparisons with DNS provides an opportunity to critically examine the applicability of the RANS models to non-laminar mixers operating at the onset of turbulence. These points are discussed using the mixing concept shown in Fig 2A, selected as the reference design, because it best achieved uniform mixing (uniform *α*) at the end of the diffuser. However, it is necessary to understand the limits of the tools used to predict mixing and the detailed physics of the mixing process for this class of dilution mixers, as discussed below.

### Turbulence models in design

The Reynolds stress tensor, given in Eq 3, is often modelled by a turbulence model. These turbulence models determine the kinetic energy *k* and turbulent viscosity *ν*_*T*_. This in turn influences the velocity and pressure fields as well as the transport of *α*. Turbulence models are based on various assumptions and as a result there is a wide variety to choose from. The impact that the turbulence model has on the final results for the reference design in Fig 2A is tested by using four different turbulence models. These models are Spalart-Allmaras [32], *k* − *ω* SST [27], Realizable *k* − *ε* [33], and the Launder-Sharma low Reynolds number *k* − *ε* [34] models.

**Fig 4:**
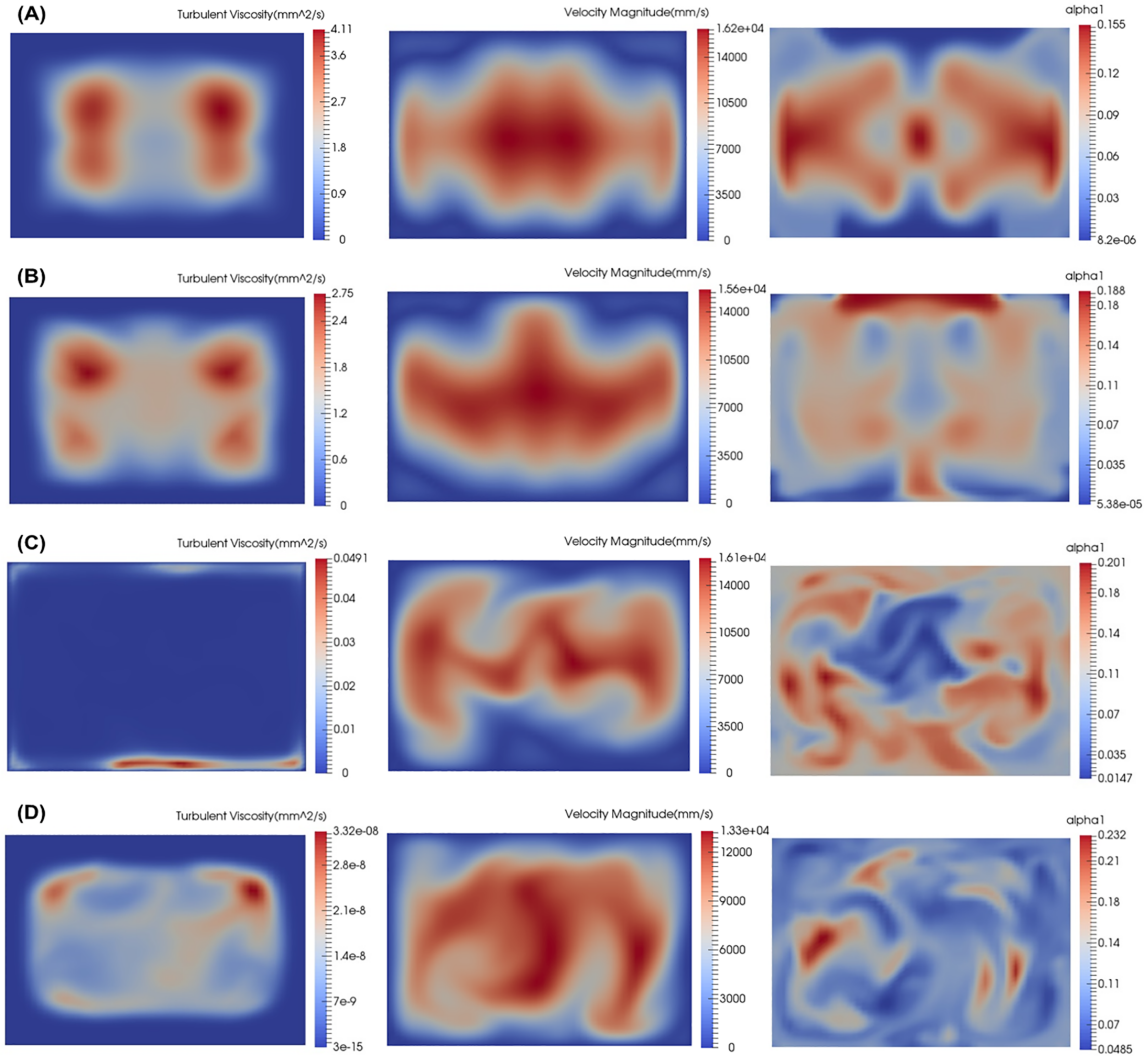
Turbulent viscosity, velocity magnitude and *α* simulation results at end of diffuser for reference design concept (Fig 2A) using various turbulence models. The position of the slices are at the “End of Diffuser” as shown in **Fig 1**. (A) Spalart-Allmaras, (B) *k* − *ω* SST, (C) Realizable *k* − *ε*, (D) Launder-Sharma low Reynolds number.

The predictions for turbulent viscosity, *ν*_*T*_, the velocity, and *α* using different turbulence models are shown in Fig 4. The turbulent viscosity, *ν*_*T*_, is a measure of where the turbulence model is active (adding turbulent viscosity to the RANS equations) and also an indication of where turbulent mixing is likely to occur.

From the plots of turbulent viscosity, *ν*_*T*_, it is apparent that only Spalart-Allmaras and *k* − *ω* SST model are active and modelling turbulence in the flow as seen in Fig 4A and 4B. The Realizable *k* − *ε* and the Launder-Sharma *k* − *ε* are not active and are not adding any significant amounts of turbulent viscosity to the flow (Fig 4C and 4D). The *k* − *ε* type turbulence models are apparently not well designed for these low Reynolds numbers (Re = 245 based on half channel width and average velocity at the mixer exit).

Of the two turbulence models that are active in this flow, the Spalart-Allmaras (Fig 4A) models the turbulent viscosity in the four corners of the channel while *k* − *ω* SST (Fig 4B) adds turbulent viscosity at the center of the channel as well as the four corners of the channel. The maximum turbulent viscosity is higher for Spalart-Allmaras, but the levels are similar. *k* − *ω* SST adds less maximum turbulent viscosity than Spalart-Allmaras, but is able to add turbulent viscosity to more regions in the flow.

The difference in the profiles for the turbulent viscosity for both active turbulence models results in mean velocity profiles that are slightly different. The Spalart-Allmaras results in a more symmetric velocity profile while the *k* − *ω* SST results in an asymmetric velocity profile. The urea concentration level, *α*, for both of these models is affected by the turbulent viscosity and the resultant velocity profile. The higher turbulent viscosity of the Spalart-Allmaras results in a lower maximum value of *α*, but the symmetric velocity profile causes a less uniform distribution of *α* across the channel. The lower maximum turbulent viscosity of the *k* − *ω* SST results in a higher maximum of *α* but a more uniform distribution.

The cases in which the eddy viscosity is not large enough (Fig 4C and 4D) show highly unsteady and 3D velocity and mixing fields. This is because the models are effectively off and the simulation is attempting to compute the model-free solution (DNS) of this flow. Unfortunately, the mesh resolution is not sufficient to do DNS properly. So these results are unreliable.

**Fig 5:**
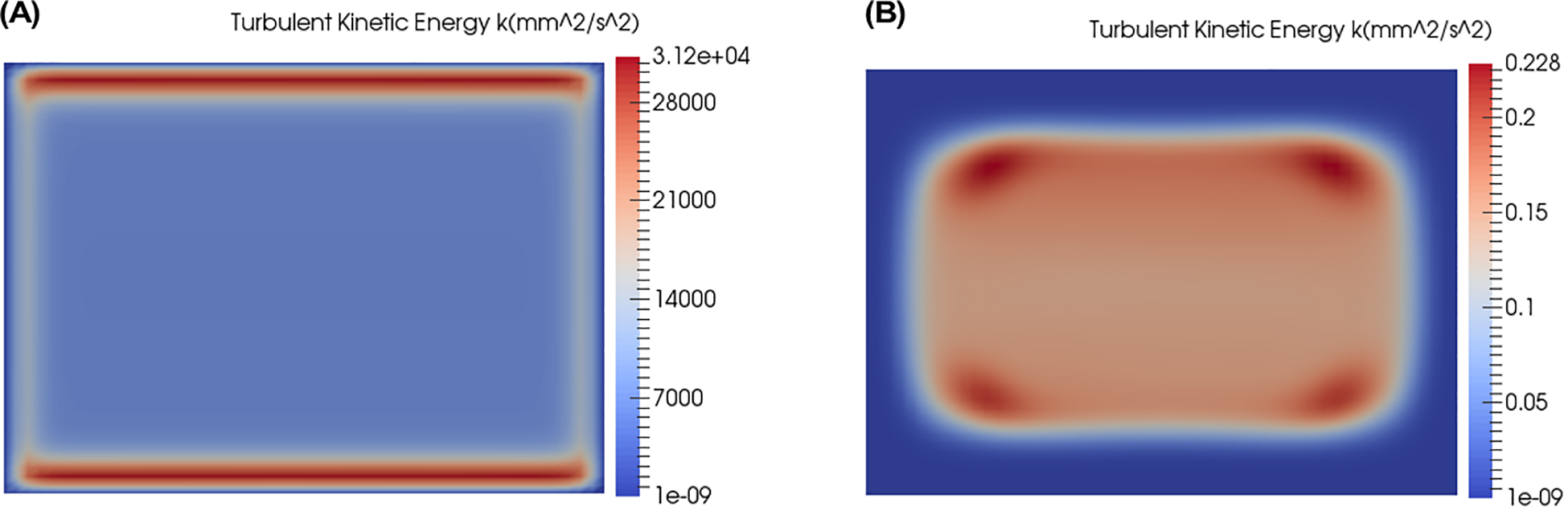
Turbulent kinetic energy k, position of slices are at the “End of Diffuser” as shown in Fig 1. (A) Realizable k-ε, (B) Launder-Sharma low Reynolds number k-ε.

Fig 5 shows the turbulent kinetic energy modelled by the Realizable *k* − *ε* and the Launder-Sharma *k* − *ε* models. This figure shows that the Realizable *k* − *ε* has a problem with the *k* equation. And the Launder-Sharma *k* − *ε* model has a reasonable *k* profile and therefore has a problem with the *ε* equation.

This application is using these turbulence models in a situation for which they were not originally designed. The models were designed for fully turbulent flows where the turbulent viscosity is at least two to three orders of magnitude larger than the laminar viscosity of the fluid [35]. However, in this class of mixers, the Reynolds number is 245 and the turbulent viscosities are only about 3 times larger than the fluid viscosity.

This study shows that the urea concentration level *α* is dependent on the turbulence model and that different models show significant variability between them. This makes it difficult to have high confidence in any of the RANS results. We therefore performed a high-resolution DNS study, discussed later, where no turbulence models are needed to fully answer the question of how mixing is occurring. Because DNS is expensive, simulations were performed only for the reference design (Fig 2A). The RANS simulations play an important role in the initial design process, but their predictions should not be relied upon for any detailed analysis.

### Mixing efficiency

**Fig 6:**
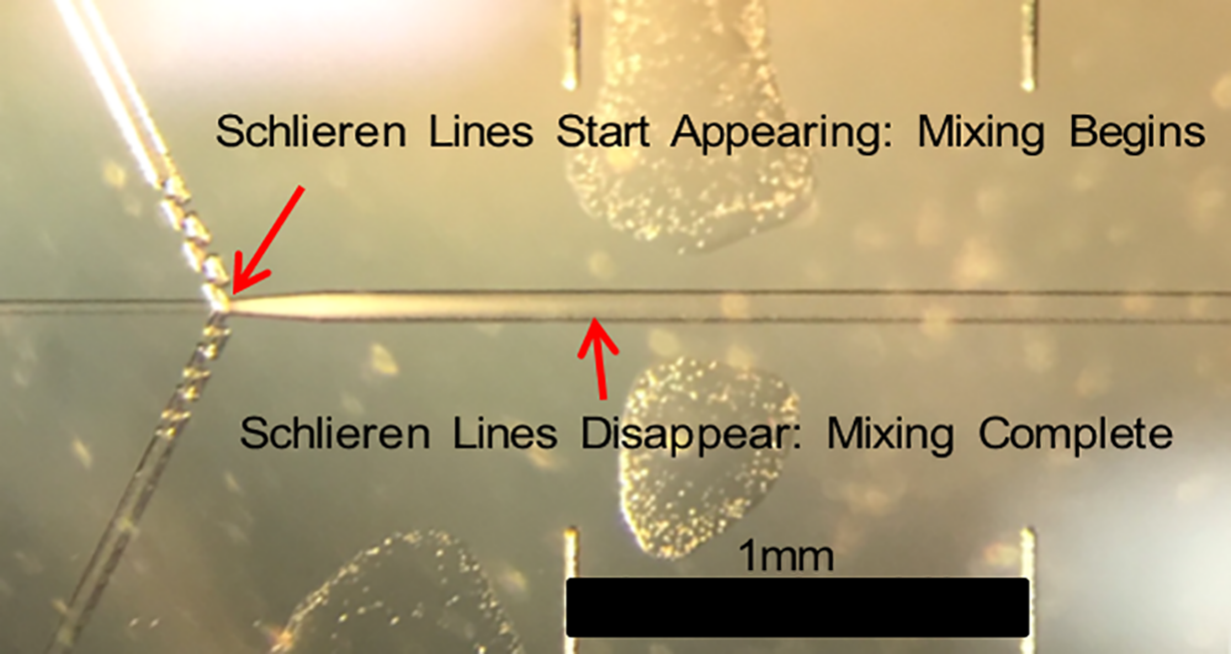
Qualitative experimental test of 10-fold dilution of 10 M urea with water visualized using Schlieren optical inhomogeneity induced by the mixing process to determine the mixing time of the reference design (Fig 2A).

Fig 6 shows a qualitative experimental mixing study performed with 10 M urea as the urea containing fluid mixing with water as the dilution buffer solution. The mixing process induces Schlieren optical inhomogeneity which disappears when the mixing is complete. From this experiment, the mixing length of the reference design can be determined and is found to be approximately 0.9 mm from where the three inlets meet. The simulated total length of the channel in our studies is 1 mm long (experimentally, the total length of the channel is typically ~15 mm). It is therefore possible to look at the profiles of *α*, and compare the RANS predictions to the experimentally determined situation (at 0.9mm from where all inlets meet).

**Fig 7:**
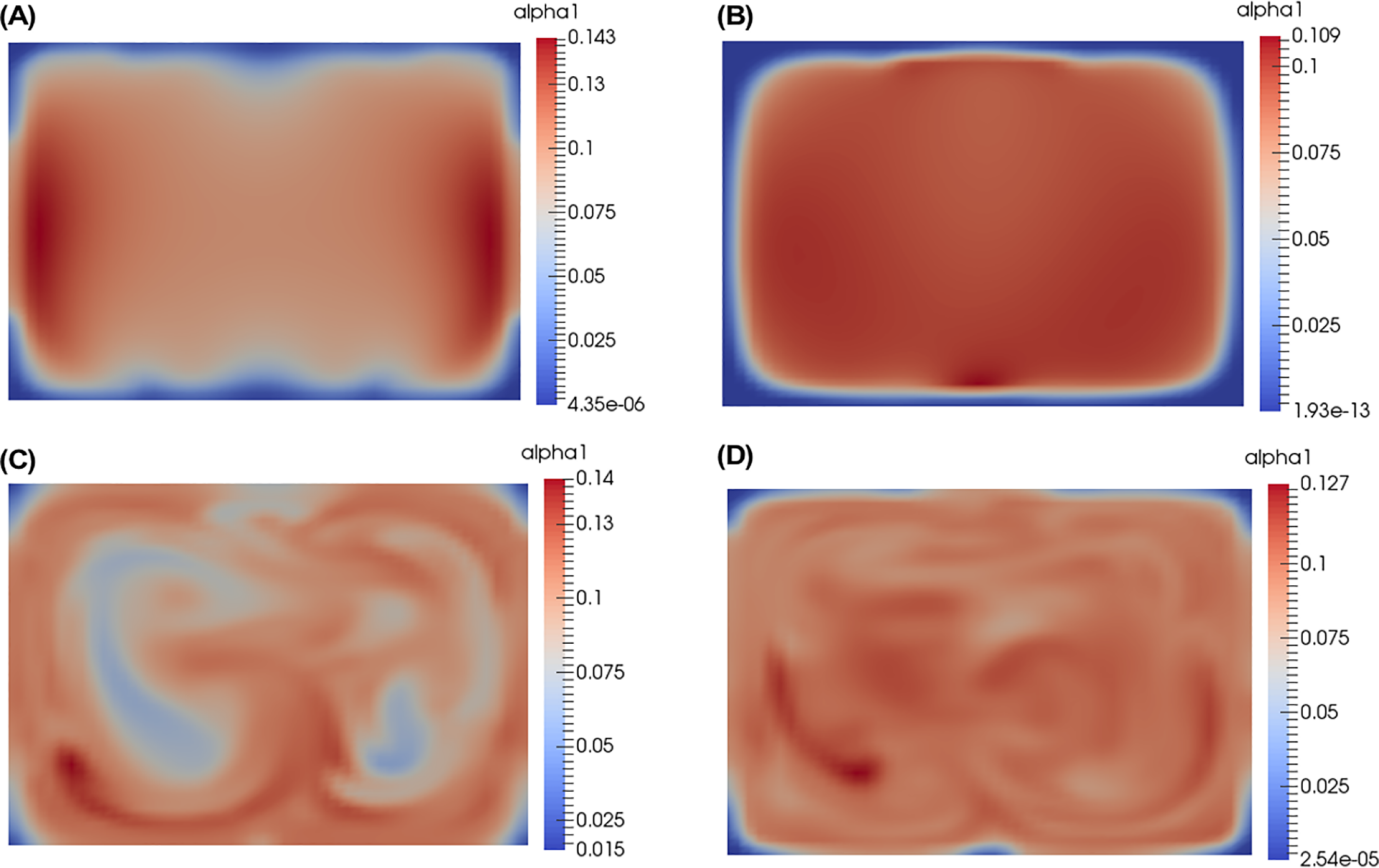
α profiles at 0.9mm downstream from the inlets. The position of the slices is shown in Fig 1 (“Fully Mixed Point”). (A) Spalart-Allmaras, (B) k-ω SST, (C) Realizable k-ε, (D) Launder-Sharma low Reynolds number k-ε.

Fig 7 shows the urea concentration levels at a distance of 0.9 mm from the intersection of the inlets. The most uniform *α* profile with the lowest maximum value is predicted by the *k* − *ω* SST turbulence model as shown in Fig 7B. The Spalart-Allmaras model (Fig 7A) also shows a fairly uniform mixing. The other two models, Realizable *k* − *ε* and Launder-Sharma low Reynolds number *k* − *ε*, (Fig 7C and 7D, respectively), where the model is not adding enough turbulent viscosity, result in *α* profiles that are uneven and unmixed. These latter two models (Fig 7C and 7D) do not agree with the experiment and are not recommended for these flow regimes.

### Direct numerical simulation

A high-resolution study was performed to remove the ambiguity presented by turbulence models and to improve the understanding of the mechanics behind the mixing process.

To resolve all scales of turbulence, the mesh resolution must be smaller than the Kolmogorov scale. This ensures the mesh is able to spatially resolve all the scales of fluid motion. The Kolmogorov length scale [36] is given by the following equation, where *ε* is the turbulence dissipation rate.

$$\eta ={\left(\frac{\nu^{3}}{\varepsilon }\right)}^{1/4}$$ (9)

To resolve the temporal scales of turbulence, the times step size taken per iteration by the solver must be smaller than the Kolmogorov time scale. The Kolmogorov time scale [36] is given by the following equation.

$$\tau_{\eta }={\left(\frac{\nu }{\varepsilon }\right)}^{1/2}$$ (10)

However, near a wall the turbulent scales are different and, *y*^+^ is used to determine the required mesh resolution. The definition [36] for *y*^+^ and the criteria [37] for determining mesh resolution from it is given by the following equation, where *y* is the distance from the wall.

$$y^{+}=\frac{y}{\nu }\sqrt{\nu {\left(\frac{\partial \langle U\rangle }{\partial y}\right)}_{Wall}}<1$$ (11)

Using the velocity gradient at the wall and the viscosity of the fluid, it is possible to determine the resolution of the first mesh cell *y* from the wall from the criteria above.

To determine the time step size which can resolve the temporal scales of turbulence the solver is set to dynamically vary its time step size based on Courant Number [38].

$$Co=\frac{U_{x}\Delta t}{\Delta x}+\frac{U_{y}\Delta t}{\Delta y}+\frac{U_{z}\Delta t}{\Delta z}<1$$ (12)

The scale of *α* is given by the Batchelor scale [39],

$$\lambda_{B}=\left(\frac{\eta }{{Sc}^{1/2}}\right)$$ (13)

So, the smallest scale of transport of the scalar *α* is determined by the Schmidt number (*Sc*) and the Kolmogorov length scale. For a urea solution with a Schmidt number of 950, the Batchelor scale is 30 times smaller than the smallest scales of turbulence determined by the Kolmogorov scales. This means that the DNS can resolve the fluid flow causing the larger scale mixing in this problem, but cannot resolve the smallest scales for the urea. For this problem, these small scales are not important to resolve.

RANS simulations of the mixer require only 2 million computational cells. Velocity resolved DNS (the results presented herein) requires 50 million cells. To resolve all the scales of the *α* would require 50 billion cells. Fortunately, it is not necessary to resolve these scales for this problem. The scalar transport of *α* simply tracks the velocity field and does not affect the development of the velocity field. The scalar field is not coupled with the velocity and pressure solvers.

The DNS simulation produces fully unsteady results. To compare to the RANS results and experiments we need to find the average flow behavior (averaged over time). This average is constructed from six instants of the DNS that were separated by a long time (separated by one device flow-through time). Ideally, more averaging samples should have been used, but limitations on computing resources prevented this, and the results are reasonably indicative even with this small averaging sample.

**Fig 8:**
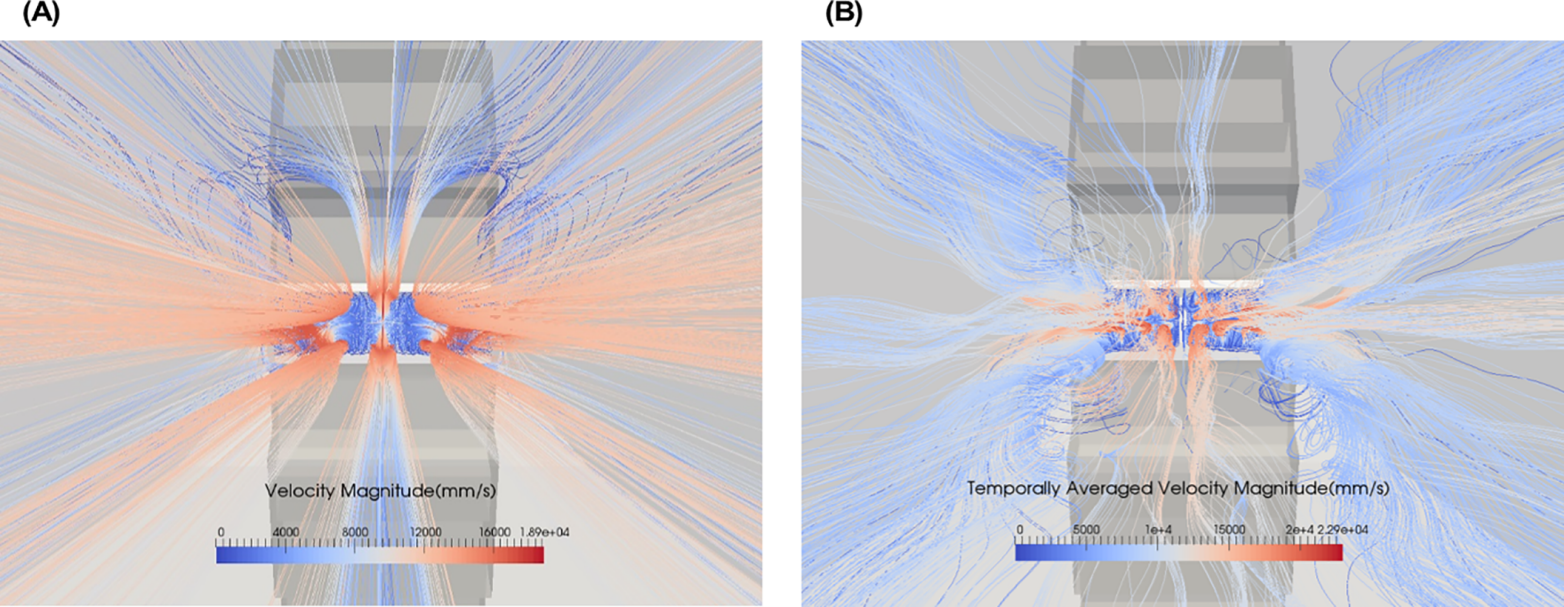
Streamlines tracking velocity and shaded by velocity magnitude in the diffuser of reference design. (A) k-ω SST, (B) Temporally averaged direct numerical simulation (DNS).

Fig 8 shows the streamlines of the mean velocity computed using (a) the *k* − *ω* SST turbulence model and (b) the averaged DNS. Comparing Fig 8A and 8B, it is observed that there still exists some unsteadiness in the DNS averages but the overall flow behavior and secondary recirculation vortices are similar for both cases.

**Fig 9:**
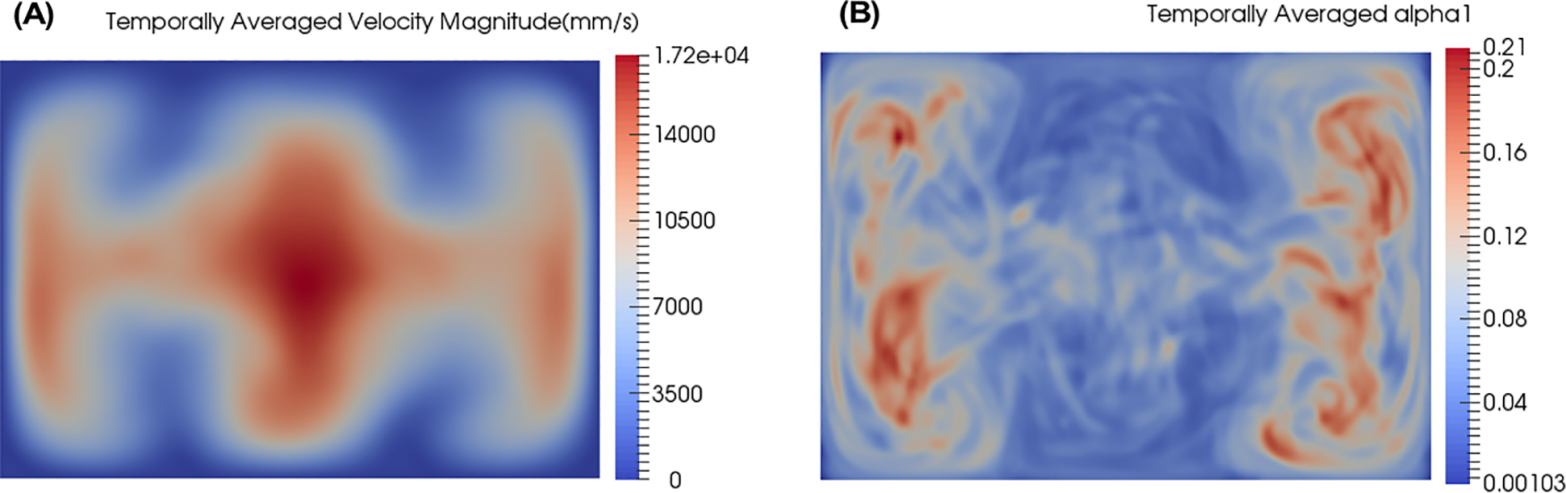
Temporally averaged DNS results at end of diffuser. The position of the slices is shown in Fig 1 (“End of Diffuser”). (A) Velocity Magnitude, (B) α.

Fig 9 shows the averaged velocity and averaged *α* profiles at the end of the diffuser for the DNS. Comparing these results to those predicted by the four tested RANS turbulence models shown in Fig 4 reveals that none of the models fully captures all aspects of the velocity and *α* profile predicted by DNS. It is interesting that the Realizable *k* − *ε* perhaps comes the closest to the DNS because it is essentially a low-mesh-resolution DNS. This indicates that using no model at all is almost as good as using a model for this type of flow. Note, however, that the absence of turbulent viscosity affects the mixing model’s ability to predict the fully mixed state (at 0.9 mm downstream) as shown in Fig 6. For the purposes of accomplishing design goals and providing easy to interpret results, the *k* − *ω* SST model may still be the better RANS model even though its predictions do not exactly match up with the DNS.

### Temporally averaged *vs*. instantaneous α

**Fig 10:**
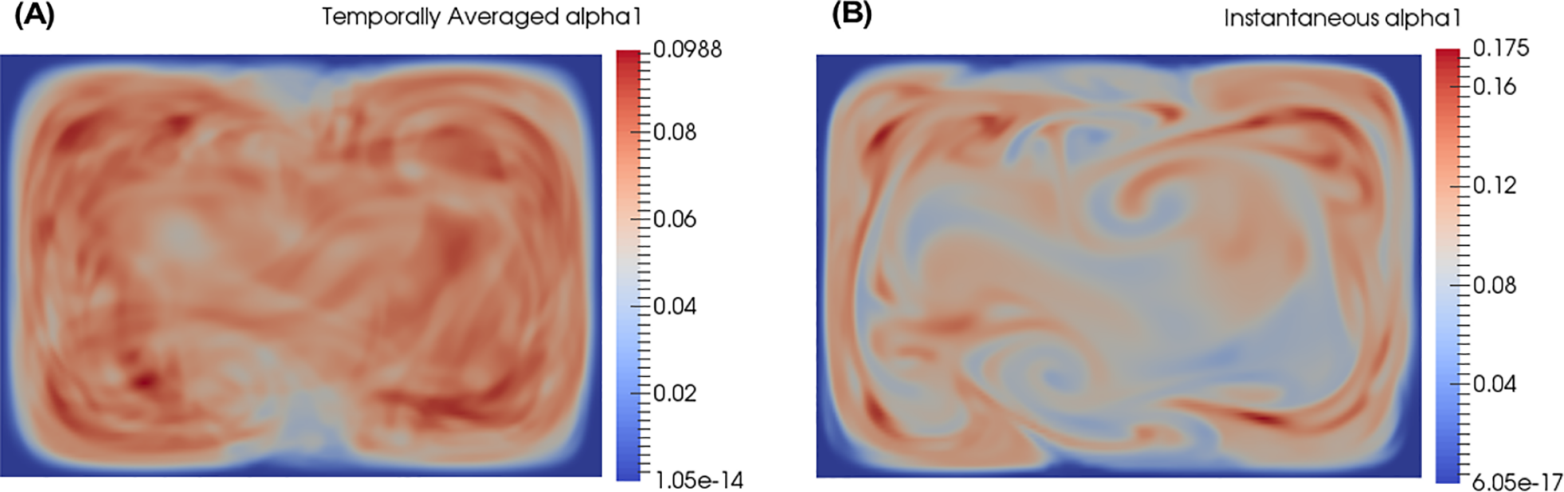
α profiles where mixing is complete experimentally (0.9mm). The position of the slices is shown in Fig 1 (“Fully Mixed Point”). (A) Temporally averaged DNS, (B) Instantaneous snapshot of DNS.

Results at the fully mixed point (approximately 0.9 mm from where the three inlets meet) are shown in Fig 10 for the DNS. Fig 10A is the time average (6 samples each at 1 flow through time apart) and Fig 10B is one of those samples. Ideally, 20 flows through time should be used to compute the time average, but this requires a prohibitive amount of computing resources. The experimental measurement corresponds to a temporal average, effectively observing the profile seen in Fig 10A. The averaging time is typically on the order of 0.5–1 s, determined by the time needed to achieve the desired signal to noise ratio. Averaging six DNS samples yields a profile that is almost even, albeit with a lot of noise due to the low number of samples used for averaging, with a value of 0.09 for *α*_1_. We expect that with more samples for averaging, the profile for *α*_1_ will approach a uniform value of 0.1.

However, when looking at the instantaneous snapshot (Fig 10B), the results show that there is significant deviation from the fully mixed value of *α*_1_ = 0.1 at any instant in time. This indicates that experimentalists should be careful about their interpretations if they are measuring highly non-linear responses. The average response may not be proportional to the instantaneous physics that is occurring locally in space and time. In Fig 10, although the average concentration of urea is 0.1, there are pockets with an instantaneous concentration approaching a factor of two higher, i.e. 0.175. This can have important ramifications for techniques sensitive to the instantaneous interparticle distance of molecules, such as SAXS.

For quick design, we recommend using the *k* − *ω* SST turbulence model and RANS simulation to design the mixer because it gives an easy to interpret profile of *α*. If a high resolution study is possible, we recommend perfoming DNS with temporal averaging using the mesh requirement dictated by the velocity and not by the passive scalar. The high cost of the DNS is not just from the larger mesh that is required, but also from the long simulation times needed to obtain sufficient averages.

## Conclusions

The mathematics behind laminar diffusion based mixing is well understood and can be done by hand without the need for computers to determine mixing times and appropriate widths for the focused solution. But incorporating more fluid physics such as chaos, turbulence, and cavitation into the mixing process requires the use of computers to design mixers in a rapid fashion to reduce the number of physical iterations and thereby the cost of mixer development. The need to add higher order fluid physics is to increase the disorder in the dilution fluid thereby reducing the mixing times and possibly reducing the bias the dilution fluid induces on the biological macromolecule. This paper presents a computational approach to facilitate the design of mixer with higher order physics.

A mixer design in which turbulence is generated prior to merging of the flow with urea is presented and shown to provide mixing in 125 microseconds at modest flow rates. This flow turning concept was able to induce the most chaos/turbuelnce into the buffer fluid compared to other designs allowing it to perform best. Though narrower channels would allow higher velocities which in turn would generate even more chaos/turbulence allowing faster mixing, narrow channels are prone to clogging and 25 μm is the lower limit before clogging becomes an issue. Comparison of our results with an experiment and DNS show that RANS simulations using the *k* − *ω* SST turbulence model can be used for early design iterations. DNS simulations reveal a stark difference between the average and instantaneous urea concentration profiles. This property of mixers relying on fluid physics other than diffusion is important to consider when interfacing with spectroscopic techniques sensitive to instantaneous concentration and interparticle distances, such as SAXS. DNS with temporal averaging is recommended as the design tool of choice for cases where details and high resolution are required.
